# The brain activity map: imaging the activity of entire neural circuits

**DOI:** 10.1186/1471-2202-14-S1-A4

**Published:** 2013-07-08

**Authors:** Rafael Yuste

**Affiliations:** 1 Howard Hughes Medical Institute, Columbia University, Biological Sciences, 901 NWC Building, 550 West 120th Street, New York, N.Y. 10027, USA

## 

The function of neural circuits is an emergent property that arises from the coordinated activity of large numbers of neurons. To capture this, we propose launching a large-scale, international public effort, the Brain Activity Map Project, aimed at imaging the full record of neural activity across complete neural circuits. This technological challenge could prove to be an invaluable step toward understanding fundamental and pathological brain processes.

